# Leadership in palliative medicine: moral, ethical and educational

**DOI:** 10.1186/s12910-018-0296-z

**Published:** 2018-06-05

**Authors:** Nathan Emmerich

**Affiliations:** 1 Visiting Research Fellow, School of History, Anthropology, Politics and Philosophy, Queen’s University Belfast, Belfast, UK; 2 Endcare Research Fellow, Institute of Ethics, Dublin City University, Dublin, Ireland; 3 The Medical School, Australian National University, Canberra, Australia

**Keywords:** Palliative, Curative, End of life care, Leadership, Morality, Ethics, Education, Philosophy of medicine, Philosophy of palliative care, The Hippocratic tradition, The Asklepian tradition

## Abstract

**Background:**

Making particular use of Shale’s analysis, this paper discusses the notion of leadership in the context of palliative medicine. Whilst offering a critical perspective, I build on the philosophy of palliative care offered by Randall and Downie and suggest that the normative structure of this medical speciality has certain distinctive features, particularly when compared to that of medicine more generally. I discuss this in terms of palliative medicine’s distinctive *morality* or *ethos*, albeit one that should still be seen in terms of medical morality or the ethos of medicine.

**Main text:**

I argue that, in the context of multi-disciplinary teamwork, the particular ethos of palliative medicine means that healthcare professionals who work within this speciality are presented with distinct opportunities for leadership and the dissemination of the moral and ethical norms that guide their practice. I expand on the nature of this opportunity by further engaging with Shale’s work on leadership in medicine, and by more fully articulating the notion of moral ethos in medicine and its relation to the more formal notion of medical ethics. Finally, and with reference to the idea of medical education as both on going and as an apprenticeship, I suggest that moral and ethical leadership in palliative medicine may have an inherently educational quality and a distinctively pedagogical dimension.

**Conclusions:**

The nature of palliative medicine is such that it often involves caring for patients who are still receiving treatment from other specialists. Whilst this can create tension, it also provides an opportunity for palliative care professionals to disseminate the philosophy that underpins their practice, and to offer leadership with regard to the moral and ethical challenges that arise in the context of End of Life Care.

## Background

In this paper I propose to discuss the conceptual particulars of moral leadership and the way in which this relates to palliative medicine as both a *clinically* and *morally* distinctive form of medical practice. To my mind such ideas turn on a specific, broadly sociological, conception of morality,^1^ understood as a – perhaps the – defining characteristic of a social field or cultural domain. This idea relates to the way in which a number of anthropologists of medicine, notably Kleinman [1] and Fox and Swazey [2], discuss the idea of medical morality, as distinct from medical or bio- ethics. They also suggest that the morality of medicine varies in differing times and places, something that also applies to differing healthcare professions, such as nursing, as well as the divisions or sub-domains of practice: the medical specialties. As is the case with these authors, I will be discussing the morality of medicine or, we might say, its *moral order* [3]. Such terms are used to refer to the normative structure of medicine, and healthcare more generally. However, rather than use the term morality, I prefer to use a slightly different concept. Thus rather than speak about ‘medical morality’ or the morality of any sub-domain, such as palliative medicine and palliative care,^2^ I tend to speak of the specific and morally or normatively defining *ethos* of a field.^3^

In what follows, then, I will be discussing the ethos of palliative care, and of medicine as a whole. More specifically, I will be discussing the way in which the ethos of palliative care apparently differs from that of medicine. A similar contrast can be found in Randall and Downie’s philosophy of palliative care [4]. In their work they set up a contrast between two medical traditions: the Hippocratic and the Asklepian. Their view seems to be that the rise of modern, professional, scientific, specialist and, perhaps most importantly, *curative* medicine has meant the Hippocratic tradition has come to dominate.^4^ They consider the inception and development of palliative care to represent a reinauguration of the Asklepian tradition in modern medical practice. Whilst I think that many of their points are well made, there is a concern that this way of looking at things has the potential to set up a false dichotomy between the Hippocratic and the Asklepian, whilst also offering an insufficiently critical perspective on medical practice, its present and its historical traditions. Instead, I suggest that both curative and palliative medicine represent a practical *realisation* of the medical ethos.

In this view the differences between curative and palliative medicine remain significant but are not without counterparts in other areas of medical practice. Consider, for example, the various ways in which the ethos of medicine, its moral order, is realised in the context of emergency medicine, general practice, and public health. Alternatively, consider the ethos of surgery, and its differential specification within commercial and non-commercial plastic surgery. Whilst similar practices may occur within each of these fields they differ in terms of their normative structure; their ethos and the underpinning values, norms, and principles that, morally speaking, define the field. There may, of course, be a significant degree of shared values, norms and principles. Nevertheless, the ethos of commercial cosmetic surgery – which has a financial motivation and is guided by certain aesthetic principles – and cosmetic surgery that, while not insensitive to comparable aesthetic principles, places health and wellbeing at the heart of its practices, and is not motivated by profit, clearly differ. Such variation is not specific to the notion of a moral ethos. Rather it is a matter of the way in which social fields are conceptualised (cf. [5, 6]). In understanding morality as a matter of a field’s ethos, a certain degree of variability must be accommodated. Indeed, one might say that the point of thinking about morality in this way is so as to accommodate the kind of variation that can be perceived both across and within particular societies or cultures. Taking this approach means that the variation we find in the respective moral orientations of palliative and curative medicine should not be seen as particularly novel. Qualitatively comparable variations can be found within both palliative and curative medical practices. Thus, whilst the difference between curative and palliative medicine may be considered marked and distinctive, there is no need to consider their differences to be as profound as Randall and Downie’s work implies. Nevertheless, their account remains informative, and reflecting on the differential influence of the Hippocratic and Asklepian traditions on contemporary medical practice and its various sub-domains or fields illuminates the understanding of the ethos of both curative and palliative medicine advanced in this paper.

Following a discussion of the notion of ethos, and what it means for the way we should understand medicine in general and palliative medicine in particular, I turn to some of Susan Shale’s ideas around moral leadership in medicine [7]. Whilst her work has focused on the management of healthcare organisations some of what she has to say about leadership in this domain applies to the clinical practice of medicine. I add to her analysis by, in particular, discussing the idea of, and need for, a specific type of leadership: that of *ethical* leadership. Part of the reason medical anthropologists have elected to focus on ‘medical morality’ has been to redirect our attention away from ‘medical ethics’ and matters that can be captured by principles, rules and codifications. Nevertheless, principles, rules and codifications are important facets of modern medical practice. Where, and when, these matters arise it can be vital for someone to take the lead, and to direct the attention of their colleagues to issues that have a specifically ethical, and not just moral, dimension.

I also intend to further Shale’s analysis by relating notions moral and ethical leadership in medicine to an aspect of both practice in general, and palliative care in particular. This is the pedagogic or educational dimension of medicine. Whilst this dimension of medical practice and leadership tends to go unremarked, it is nevertheless commonplace to suggest that the process of medical education is one of an extended apprenticeship (cf [8, 9]). As such it is not completed on graduation from medical school, and nor should we presume it is complete when one finishes foundational training. Furthermore, in an era of Continuing Professional Development (CPD), we need not presume that it has been completed at some point prior to becoming a consultant or a partner in a GP surgery, say. Given the current culture of medicine, the imperative towards multidisciplinary team working in end of life care, and in the context of the on-going nature of professional education, I wish to suggest that those who work in palliative medicine and, one might add, palliative care more generally will often find that they encounter circumstances that have a pedagogical component or value. Somewhat simplistically, I think that they will find that, in the course of normal practice, ‘teachable moments’ are not uncommon. Some of these moments will be fairly explicit in nature; they will involve the opportunity to question norms and suggest alternatives. Others will, I think, have a more implicit nature and will require palliative care professionals to act as exemplars or role models, and, in so doing, to promote emulation amongst others, including non-palliative care specialists. It is in regards these latter pedagogical endeavours that the issue of professionalism will come to the fore.

## Palliative care as/and the ethos of medicine

Whilst I think that most of us would have an intuitive grasp of what is meant when someone speaks of the ethos of medicine, the concept of ‘ethos’ itself has been the subject of analytic neglect. With the exception of two essays by Wolff sustained analysis of the notion is, for the most part, absent [10, 11]. This may well be due to the flexibility the term exhibits; something that analytic philosophy and applied ethics often takes as prima facie indication that a particular concept is of little intellectual use. However, in the first instance, my use of the term can be situated within the context of a broadly Bourdieuan social theory. In this context the fact that the term can be applied to both social fields – the ethos of medicine - and individuals – as in the dispositions of (moral) character – is a strength. For Bourdieu, dispositions of habitus and the social structures of fields are homologous or ontologically complicit [12]. Given this entanglement the fact that the term ethos can refer to the characteristics of individuals (habitus) and social fields should not be seen as being essentially problematic in nature.

Nevertheless, I use the term as a field level concept and to refer to the normative social structure, moral shape or *order*, of particular fields. Unfortunately, this raises another difficulty. Social fields do not have a singular existence, but can be realised and attended to at a variety of levels, depending on the focus of one’s attention [5, 6]. Consider, for example, the ethos of a national political party, and the potential differences between this and the ethos of its local branches. The normative structure of the larger body does not simply determine those of the smaller, subsidiary, bodies. Equally, the normative structure of the larger body is not simply an aggregation of the smaller, constitutive, bodies. Similar thinking can, for example, be applied to the UK’s National Health Service (NHS) and the institutions - hospitals, primary care trusts, GP surgeries, the National Research Ethics Service – that can be located within it. In this light it may be that the term ethos has been neglected because it seems unable to capture a singular moral order in contexts that we assume one should exist. However, this seems less of a problem for ethos and more of a fact about social reality. As such, it is out presuppositions that seem misguided. There is an irreducible complexity to social ontology both in terms of scale (considering the world at different levels) and in terms of the boundaries we consider to define the limits of particular social fields. For example, consider whether or not the various Royal Colleges of the UK’s healthcare professions are part of the NHS, and whether or not the ethos of the NHS can be considered as an important part of their moral character. To my mind the answer the former question is no, whilst in relation to the latter question it would seem to be yes.

In the light of these comments we can consider the connection between palliative medicine, other medical specialties and medicine as a whole. Whilst it is arguably the case that all specialities have their own distinctive moral orders, it is equally arguable that there is something specific about that of palliative medicine (and – and as a part of - palliative care) when we consider the relationship between these moral orders and that of medicine as a whole. Randal and Downie’s philosophy of palliative care can be taken as suggesting that this is due to the fact that it is informed by an alternative medical tradition [4]. Their view is that palliative care is Asklepian whereas other specialities, and medicine as a whole, are Hippocratic. However, their analysis seems to suggest that the relationship between these two traditions has a binary nature; that there stand in a dichotomous relationship. As such it would seem that the influence of the Asklepian tradition on palliative care precludes the relevance of the Hippocratic tradition, and vice versa. Thus, the pre-eminence of the Hippocratic tradition in modernity entails the exclusion of Asklepian values. Such a view would seem to suggest that the moral order of palliative medicine – and palliative care more generally - not only significantly differs from other areas of medical practice, but that they are incompatible with one another. It would be better, I think, not to adopt such a radical point of view and, instead, take note of Frist and Presley’s comment on the WHO definition of palliative care:

"The World Health Organization defines palliative care as:

“care that improves the quality of life of patients and their families facing the problems associated with life-threatening illness, through the prevention and relief of suffering by means of early identification and impeccable assessment and treatment of pain and other problems, physical, psychosocial, and spiritual.”

*This should be how all medical care is defined*."

William H. Frist and Martha K. Presley [13] “Training the next Generation of Doctors in Palliative Care Is the Key to the New Era of Value-Based Care.” *Academic Medicine.* Italics added.

In an era of high tech medicine the possibility of a cure has, it seems, come to overshadow what was once a central part of all medical care. The success of scientific medicine during the late 19th and early 20th centuries, the social consequences of the cognitive (re)structuring of medicine into specialisms, and the emergence of nursing as a distinct profession, are such that the importance of the kind of care highlighted in the WHO definition of Palliative Care has been obscured when it comes to medical practice in general. In cases where the possibility of a cure begins to recede it once again possible to recognise and to realise (to *re-cognise* and to *real-ise* or *render real*) that professionals need to be able to provide patients with this sort of care. Among the first to do so was Cicely Saunders.^5^ The fact that her career was dedicated to caring for terminally ill oncology patients is, of course, highly relevant. This is a field where days, weeks or months prior to the death of a patient, the curative treatments on offer can become exhausted. It is also a field where curative treatment itself can be the cause of high levels of discomfit and distress. It is also a context that not only lends itself to the involvement of the patient’s family in patient care, but one that gives rise to a high degree of distress among all those involved with the patient and their care. Given broader cultural changes in which religiosity is in decline, and both death and bereavement are increasingly medicalised, the factors mentioned can all be seen as contributing to a reassessment of how healthcare professionals could meet the needs of terminal – dying - patients and what it might mean to provide care at the end of life. Doing so did not involve abandoning the ethos of medicine but looking to it for resources appropriate to the needs dying patients and the families of such patients.

In my view the notion of ethos allows us to picture these resources as well as to accommodate the Hippocratic and Asklepian medical traditions in a manner that does not risk representing them as opposed or dichotomous, or as in some way competing or conflicting, ideals. As suggested, an ethos can be realized (or *rendered real*) in a variety of ways or, to put it another way, different aspects of the same ethos can come to the fore (or fall into the background) depending on the context, and the scale at which we elect to focus our attention. Palliative care is not only part of modern medicine, but draws on and develops its historical and normative traditions. As compared to other medical specialties, the demands of palliative care are such that different facets of the medical ethos are called forward, whilst other aspects tend to be relegated. The fact of specialization – and the associated modes of social organization of practice, including training, and medical apprenticeships - has meant that these differences have been concretized. Whilst this may once have been productive, and allowed for the establishment of the field of palliative care as well as palliative medicine as a distinct specialty, it is possible that such specialization is now becoming unhelpful. Furthermore, explaining this difference in terms of opposing or contrasting medical traditions is an appealing strategy. As such the existing structural arrangements seem to be given sense by Randall and Downie’s position; it can appear as if palliative care and curative care exhibit basic or fundamental differences. Nevertheless, an appeal to unity has more to recommend it, even if that unity must accommodate no small degree of diversity.^6^

It is clear, then, that the notion of ethos offers a distinctively pluralist perspective on the normative structure of social fields. Curative and palliative medicine sit alongside one another and, in the final analysis, are part of the same cultural phenomena: modern medical practice. However, whilst neither denies the other, both curative and palliative medicine have differing priorities when it come to the aims, objectives and goals that should be pursued in practice. From the perspective of curative forms of practice it will likely be justified to sacrifice a patient’s quality of life in both the short and the medium term. In contrast, a palliative approach invites us to reconsider this pact. It may be that an increase in the patient’s quality of life may justify forgoing treatment that has the potential to increase life. At minimum, the ethos of palliative care is such that it promotes the reconsideration of the purpose of medical treatment, and promotes the provision of supportive care in such a way that the patient can pursue their own ends in – or with - the time that they have left. This does not, of course, necessarily mean forgoing any and all forms of curative or life-prolonging treatments; palliative care can be provided in tandem with life-prolonging treatment and, at its best, can itself prolong life as well as aim at promoting its quality. Thus, some may wish to receive treatments that will negatively impact their quality of life in order to remain alive for an anniversary or other event to which they attach importance, significance or meaning, whilst others may wish to emphasize their quality of life over a shorter period. This can be seen as a facet of a broader difference. As Randall and Downie suggest:

“[I]n conventional health care psychosocial and spiritual care is not primarily undertaken by health care professionals – and indeed is not seen as part of healthcare at all. But palliative care emphasizes that psychosocial and spiritual care are part of the remit of healthcare professionals, possibly because they are thought to contribute to quality of life, and to one ideal of a ‘good death’. Here, again palliative care is different from conventional health care” [4].

To my mind, it is better to take a slightly different perspective; one that takes psychosocial and spiritual care to be part of medical practice in general, whilst also acknowledging that different forms of medical practice involve differing kinds or degrees of psychosocial and spiritual care. This view seems better able to accommodate the activities of various healthcare professionals, including general practitioners and those who work in nursing homes, for example. Perhaps, then, the difference between palliative and conventional or curative medicine would be better characterized as follows:

Curative medicine seeks to *return* patients to their own lives, so that they might *independently* pursue their own priorities and do so in the manner of their choosing. As part of palliative care, palliative medicine seeks to *assist* patients in leading their lives and to *support* them in the pursuits they wish to prioritize in the time that they have left.

This is, then, an important facet of the ethos of palliative care. The focus is on the person as a whole, it is holistic and encompasses - and seeks to support - the patient’s own ends. In taking on this role the provision of palliative care often entails supporting the patient’s family in a manner that is of more immediate or direct relevance than is the case in medical practice more generally. Rather than seeking its elimination, as part of palliative care palliative medicine involves managing the patient’s medical condition(s) and associated symptoms. This is clearest in end of life care. In general, the prevention of death can be taken as the raison d’être of medical practice, and the notion that someone is dying is taken to be a corporeal fire alarm, or as marking out the timeframe in which doctors have to complete their work. In contrast, palliative care accepts death and dying as part of life, and seeks to ensure that those who have reached this point in their lives are as comfortable as is possible, and, insofar as is possible, are able to pursue the actions and ends they consider important.

### Moral leadership in palliative medicine

Given the analysis presented in the previous section we can conclude that those who specialise in palliative care are involved in the pursuit - or realisation - of a specific form of the medical ethos. Whilst there is, at the present moment, a general tendency to think that everyone should be exhibiting some form of leadership at all times, it is important to recognise that healthcare professionals can pursue the realisation of the medical ethos with out necessarily doing so as leaders, or whilst exhibiting something called leadership. That said, if the kind of education discussed below can be considered a form of leadership, then it might be possible for palliative care professionals to act as leaders whilst doing little more than fulfilling their normal, everyday roles and responsibilities. In a similar vein, some definitions of leadership are such that leadership need not be seen as a specific kind of activity but rather are about the way in which some are able to conduct themselves. Consider, for example, the way Shale conceptualises moral leadership in medicine, which she considers it to be “the process of orchestrating organisational moral narratives” [7]. Clearly, those who are not suitably situated within an organisation cannot accomplish such orchestrations. As a result, the kind of leadership an individual can pursue is closely linked to the location they occupy within particular social fields.

Given Shale is discussing the moral leadership of medical managers, whereas the present discussion is focused on moral leadership in the context of clinical practice, the notion can be recast as *the process of orchestrating moral narratives that guide the treatment and care of patients*. Such narratives certainly have an organisational dimension but one that is less a matter of the *institutional* organisation that concerns Shale. The kind of organisational leadership exhibited by healthcare professionals who are acting as such relates to the way in which clinical practice is structured. This might be a matter of organising care for individual patients or might be more general and related to the provision of care more generally. As such, we should not think of the difference between clinical practice and the organisational endeavours of management as involving any kind of clear or absolute distinction. Nevertheless, even as we build on the insights offered by Shale, we should not presume that her work can simply be appropriated to the task of conceptualising moral leadership in clinical practice. As she suggests, the nature of moral leadership may differ from one context to the next and the kinds of moral leadership exhibited by managers of healthcare organisations may differ from that offered by professionals in the course of their clinical practice [7].

In her work Shale suggests that leadership involves broad, general practices of responsibility and articulates a notion of propriety as a way to specify or identify more concrete forms of such practices. In the context of medical management, she identifies five specific kinds of propriety, these being: fiduciary; bureaucratic; collegial; inquisitorial; and restorative. Whilst the practice of palliative care may involve bureaucratic, inquisitorial and restorative propriety they do not directly relate to moral leadership.^7^

Fiduciary propriety involves the principle that a doctor’s first priority must be attending to the needs of their patients whilst collegial propriety is “a way of behaving suited to an enterprise in which participants rely not upon hierarchy, but upon goodwill and cooperation, to meet their professional and moral responsibilities” [7]. Given the nature of palliative care the fiduciary propriety governing its practice not only significantly differs from that of medicine more generally it may also come into conflict with the fiduciary propriety of others. Whilst both have fiduciary responsibilities that require them to prioritise the needs of their patients, the general presumption that guides the care offered by medical professionals is that what the patient needs is curative treatment. Palliative care professionals need not reject this notion. Rather it is the case that they recognise the potential for curative treatment may be limited and that what their patients need may not solely relate to biomedical matters, strictly defined, but to other areas of their life. In such cases, doctors from differing specialities must enter into dialogue with each other in an attempt to produce a coherent moral narrative that might guide the treatment and care of the particular patient at hand. Given that palliative medicine can be considered a relatively heterodox form of medical practice, and that medical professionals are no less susceptible to the denial of death than patients, when it comes to providing care at the end of life, palliative care professionals must work the hardest to orchestrate the operative narrative, the one that is actively guiding the care of the patient. Given the collective and team-based nature of medical practice in this area, such orchestration will be fundamentally dialogical and involve the mutual exchange of views and perspectives on the kind of care appropriate to the patient(s) at hand. In this context leadership does not mean imposing one’s view but successfully presenting it to others in such a way that its validity can be recognised.

Such thinking naturally leads to a consideration of Shale’s notion of collegial propriety. Given the above comments there is significant potential for palliative care professionals to find themselves in a position of challenging the dominant narrative of patient care. It is difficult to challenge the orthodox way of doing things, the prevailing ethos or, we might say, the established moral order. This will be particularly relevant in places where palliative care has not yet been established. Whilst it can be frustrating, and whilst it can feel as if one is failing to discharge one’s duties to one’s patient, operating in a manner that respects established norms of collegial propriety is a reflection of leadership. Whilst this should not be taken to mean that collegiality must be maintained at all times, or that leadership can never involve imposing a point of view, leadership is better understood as the ability to bring people along with you, to render others as fellow travellers. The ability to maintain collegial relationships with other professionals is, clearly, an important part of such leadership. Furthermore, such collegiality is essential to the broader establishment of palliative care both alongside curative care and, perhaps more importantly, during the transition from the latter to the former. Such a transition involves moving from one ethos to another, and allowing certain values that inform medical practice to move into the background whilst other values come the fore.

### Ethical leadership and palliative medicine

When Shale speaks of the morality of medicine or moral leadership in medicine she has in mind a broad, structural and relatively formal or, at least, explicit, conception of the normative dimension of social life. Consistent with their use in both everyday and professional discourse Shale considers the terms morality and ethics to be “almost but not quite interchangeable” [7]. As noted above, my conception of ethos is related the anthropological idea of medical morality. As such it is used to distinguish between the socio-cultural normativity embedded in practice and a more formal notion of (medical) ethics that is embedded in the discourse of applied (bio)ethics and professional codifications. Nevertheless, there is potential for a theoretical reconciliation of the more diffuse and tacit normativity that structures social fields and acts as the operative logic of practice – i.e. the morality or ethos of medicine - and the notion of ethics as the more formal, explicit and codified phenomena that can be articulated and reflected upon by social actors. Such ethics, their formal articulation and cognitive or reflective role are not mere epiphenomena. Nor are they fundamentally distinct from practice. Rather, both reflection and the articulation of ethical values, norms, and principles are specific forms of (cognitive) practice and can be embedded in practical activities, such as clinical medicine, or in intellectual activities, such as the kinds of ethical reflection we find in ethics committees, medical ethics classrooms and the activities of academic ethicists, including those of philosophers. Regardless of the origin of such codifications - i.e. given the case at hand, medical ethics, whether or not they are the confabulations of applied ethicists and philosophers, the product of socio-historical processes of professional organisation, or, as is the case at the present moment, some admixture of the both – any ethics is always rooted in the practices, and therefore ethos, of one (or more) field(s).^8^

Given the complexity of the notion of ethos, the relationship between it, and the articulation of any substantive ethics, is not simple. The ethics of a particularly field of practice, medicine say, is not simply defined by its ethos or determined by the normative social structures associated with the field. Furthermore, an ethics may be influence and shaped by the ethos of an external field. This can be perceived in the case of modern medical ethics, something that has clearly been influenced by applied (bio)ethics. As this suggests, over time an ethics can contribute to the reformation of a field’s ethos and, thus, an ethos can be influenced by external fields and practices. This often occurs through ethical discourses, exchanges and commentaries, but can also involve deeper and subtler socio-cultural processes. For example, the hand of (bio)ethics can certainly be discerned in the development of patient autonomy and, therefore, medicine’s repudiation of paternalism. However this development might also be related to the advent of consumerism in medicine. The fact that changes to the doctor –patient relationship can be understood as reflecting broader social norms indicates that there is more too patient autonomy than the advent of biomedical ethics. Nevertheless, given the issues at hand, such broader considerations can be left to one side. If it is to be understood properly, the ethos of medicine can and should be situated in and related to the broader moral context. However, for our present purposes it is enough to recognise that an ethos of social fields like medicine and is sub-specialities can, more or less directly, be influenced and developed by more reflective discourses like medical and bio- ethics.

We might say, then, that the relationship between medical ethics and the ethos of medicine is, co-productive [14]. The ethos of medicine provides a normative context for the articulation of medical ethics and, overtime, formal medical ethics can contribute to the reformation of the medical ethos. In this view, medical ethics education can be seen as an influential conduit, effecting change and promoting medicine’s transition from what was an ethos of paternalism to one in which patient autonomy is given greater priority (cf. [15]). Finally, and more pertinently, given that the ethos of an *overarching* field can take on differing shapes in distinct sub-fields – albeit differing shapes that share a certain family resemblance - then we might expect to find that differing ethical imperatives are accorded differing priorities, or understood in a slightly different manner, within different sub-fields. To my mind this is what we find in the case of palliative medicine as compared to other medical specialties as well as medicine as a whole. Indeed, although the contrast is, perhaps, not as great, similar thinking can be applied to palliative care with regard to healthcare as a whole. In this context we might, then, consider the notion of *ethical* leadership in the context of practicing palliative medicine.^9^

The nature of palliative medicine is such that those working in this field commonly encounter ethical issues that are relatively uncommon in other areas of medical practice. This includes, for example, the increased use of pain relief, possibly to the point of ‘terminal’ or ‘continuous’ sedation. It may also include the withdrawing or withholding of life saving or life prolonging treatment. This can include Cardio-Pulmonary Resuscitation (CPR), artificial ventilation, Artificial Nutrition and Hydration (ANH) and using antibiotics to combat an infection such as pneumonia. To some extent there seems to be an acceptance of Do Not Attempt Resuscitation (DNAR) notices and, therefore, with the idea of withholding treatment. Nevertheless, there is a broad reluctance to withhold CPR from those who have not consented to such notices.^10^ Amongst other things this has, it seems, created the conditions where CPR is delivered to a large number of patients who are not only dying at that moment but who are a. in the final stages of terminal illness, and b. unlikely to benefit from what is a fairly invasive and violent intervention. In this context, there is a clear potential for palliative care professionals to pursue a leadership role by raising the matter of whether or not procedures like CPR are futile and should therefore be withheld.

This point can be applied more generally. Palliative care professionals are well placed to question the introduction or maintenance of treatments that may no longer have the potential to benefit patients or, in the case of interventions with significant side effects, which may no longer meet the threshold of providing an overall benefit to the patient. In this context raising questions about whether or not it is appropriate to continue or discontinue treatment can be a form of ethical leadership. This remains the case whether the treatment at hand is ANH or life support, if it is long-term medication for a pre-existing condition, or if it concerns the provision of, say, antibiotics in response to a recently acquired infection. The impetus to raise these questions, and the inclination not to consider them, is rooted in the respective ethos of palliative and curative medicine. However, raising them is one small part of the process. What is important in these case is to give due consideration to the matters at hand from both a clinical as well as an ethical perspective. Johnston, Cruess and Cruess suggest “[e]thical leadership entails leading others in setting standards or, and therefore defining, moral or acceptable behaviour” [16]. The ability to provoke, structure and lead such conversations is a matter of ethical leadership and whilst such conversations should not result in the imposition of a ethical narrative by palliative care professionals, it is legitimate to think of such discussions as, pace Shale, involving a process through which the narrative of patient care is being *orchestrated*.

The effect of palliative care professionals pursuing such leadership activities as the ones I have discussed in this section clearly has the potential to promote ethical practice as well as to impact positively on patient care at the end of life. However, more than this, ethical leadership has the potential to effect broader reforms on the ethos of medicine. As Brodwin suggests, ethics and morality (ethos) stand in a relationship of mutual co-production and reproduction [14]. Furthermore, in the above-cited quote, Frist and Presley suggest that the substantive definition of palliative care offered by the WHO should be understood as defining medical care more generally [13]. Given the comments offered by Randal and Downie and my reinterpretation of the way they present the Hippocratic and Asklepian traditions, it would seem that palliative care brings to the fore something that, in its singular pursuit of a cure, modern medicine has a tendency to neglect [4]. The view I have set out is that certain of medicine socio-cultural values are central to the practice of palliative medicine. These values are not entirely absent from medical practice more generally; rather, it is the case that they are somewhat marginalised and relatively peripheral. Returning them to the fore, in a manner that can engage medical professionals in a discussion of the ethical dimension of end of life care, is an essential facet of leadership in the care of dying patients. Understanding that such discussions are not a matter of reasoned exchanges alone, but involve an encounter between differing realisations of the medical ethos, is essential to good leadership in this domain.

### Education and the practice of palliative care

In the previous sections I discussed ideas of moral and ethical leadership in the context of palliative care. In this section I suggest that such endeavours have an inherently pedagogical dimension. This pedagogy is of a certain kind, it is a largely tacit phenomena and, rather than involving the simple *acquisition* of explicit knowledge, it suggests changes and developments in practice that occur as the result of frameworks of *participation* [17]. Such perspectives are rooted in anthropological conceptions of socio-cultural learning theory as well as associated notions of situated learning and ‘apprenticeship’ [18–20]. I have previously made use of such theoretical accounts to sketch a connection between the moral socialisation of medical students and what I call their ethical enculturation [21]. Whilst such work informs the following discussion, for current purposes it is perhaps better to focus on the notion of emulation and role modelling [22]. In so doing one can promote the idea that the kind of leadership discussed above involves palliative care professionals acting as exemplars of medical morality, and that this can prompt others to emulate their actions. In this way one can perceive such actions, and such leadership, as offering an implicit pedagogy to those who might follow such leadership.

Whilst it is clear that reflective debate and the reasoned exchange of views is essential to good medical practice and end of life care it is also the case that differing ethical perspectives can be rooted in differing moral orientations or ethos. Similarly, whilst it is certain that the underpinning moral order or normative social structures of medical specialities, and the embodiment of them by individuals in practice, are not fixed they are, without question, highly durable. Furthermore, given the fact that the ethos of medicine influences the reflective practices of professionals – the particular ways in which they think and reason – it is not entirely possible to think of healthcare professionals as subjecting it to independent analysis. Whilst it directly conditions practice through its embodiment in the dispositions of habitus, as an aspect of a social field any ethos has a largely implicit or tacit existence. Our sense of the moral order of the field(s) in which we are located is acquired over time, and a function of an individual’s exposure to practice and associated process of habituation; it is produced through interrelated processes of socialisation and enculturation [21]. Given that any analysis of the ethos one inhabits inevitably involves moral considerations, then one cannot but bring to bear one’s moral point of view, something that is fundamentally formed and informed, shaped and reshaped, by the ethos one inhabits.

Whilst this places limits on the way in which we should understand the moral point of view,^11^ no ethos is entirely uniform. In the preceding discussion I have, for example, held that the ethos of medicine is variously realised in different medical specialities. In this context, one route through which palliative care professionals can educate other professionals from other specialties is by simply pursuing their professional responsibilities and discharging their duties in an exemplary manner. In so doing they can provide a powerful demonstration of palliative care’s ends and the value it has to offer patients, their families, and the healthcare system as a whole. If, as they pursue their work, they can also externalise their evaluation of the case(s) at hand, then such demonstrations are likely to be more effective. Such externalisation is not the relatively simply task of expressing one’s thinking about the case at hand, but the broader task of expressing one’s thinking whilst also giving others a sense of the underlying perspective; the orientation that informs and underpins the clinical evaluations that one has to offer. As such, both the reflective practices and the clinical practice of palliative care professionals can transmit, or make available, the particular values of the field of palliative care. To practice in this way is to present oneself as a role model. One need not, of course, be explicit about this aim. Our social norms are such that holding oneself up as an exemplar is rarely a productive strategy. Nevertheless, the pursuit of leadership can legitimately entail a conscious attempt to act as a role model.

Such thinking about the educational possibilities presented to palliative care professionals is a little different to the idea that one might try to be aware of ‘teachable moments.’ Whilst not denying that such moments may arise, they are somewhat limited. For better or for worse, the normative structure of medicine is marked by a certain degree of hierarchy. The notion of a truly teachable moment is no less subject to this hierarchy that any other facet of medical practice. One can take this as an indication that such moments may arise between palliative care professionals and medical students, those completing foundation years and some doctors at an early stage in their careers. Nevertheless, they are likely to be uncommon in other contexts. Furthermore, the palliative care literature contains some suggestions on specialist education. Such work often promotes the view that encounters with dying patients can be sources for the development of the correct moral attitude [23]. Such encounters should, of course, be accompanied by the kind of reflective practices that are now central to education and practice in both medicine and healthcare more generally [24, 25]. It seems, then, that medical students and healthcare professionals in general can draw on their experiences in the field of palliative care and, in particular, their encounters with dying patients to develop as empathic and caring professionals [26–30]. Those working within palliative care would then be well advised to encourage such encounters and to facilitate the subsequent reflection of others. This may entail little more than providing a sympathetic ear, allowing colleagues the space – or, simply, offering permission – so that they might discuss their experiences to whatever degree they find necessary or helpful. Whilst the promotion such activities do not directly meet Shale’s criteria of leadership – the orchestration of moral narratives – they can be though of as making an indirect contribution to the way in which the healthcare professional concerned will shape such narratives in future.

## Conclusion

Whilst palliative medicine – and palliative care more generally - differs from medical practice it is, nevertheless, part of this broader enterprise. As such we should understand its morality or ethos do be an instantiation or realisation of the ethos of medicine. Adopting this point of view we can appreciate that the values, norms and principles that come to the fore in both palliative medicine and palliative care are not absent from medical practice more generally. It is merely the case that each has a different emphasis. Some of the values, norms and principles that we find in the forefront of curative medical practices do not receive the same emphasis in palliative contexts. There is then, a certain degree of commonality between palliative and curative medicine, as a result there should be room for an appreciation of the shared aspects of their respective ethos. Nevertheless, one should acknowledge that palliative care is a subaltern medical culture. Whilst this may cause it to be somewhat neglected, or to be relegated to ‘Cinderella’ status, this does provide its practitioners with opportunity for moral, ethical and educational leaderships. Particularly in the context of an aging population, and increasing levels of chronic, and often terminal, illnesses amongst that population, it is becoming increasingly clear that what palliative care has to offer is what many patients require. Furthermore, at its best, palliative care can both improve patient’s quality of life and its quantity or length. Whilst any number of editorials and op ed. pieces can state these claims, the best proof is to be found by demonstrating the benefits of palliative care; displaying the contribution palliative care can make to patients is the best route to being involved in the orchestration of treatment more generally. Over the past few decades palliative care has established itself as a legitimate medical speciality. The challenge it now faces is to maintain this status whilst also become embedded in, or available to, medical practice more generally. One route to meeting this challenge is through providing the moral, ethical and educational leadership considered above.

## Abbreviations

ANH: Artificial Nutrition and Hydration
CPD: Continuing Professional Development
CPR: Cardio-Pulmonary Resuscitation
DNAR: Do Not Attempt Resuscitation
NHS: National Health Service

## Footnotes

1 Sociological and anthropological studies that seek to understand the social and cultural reality of morality and ethics have, after a long period of absence from these disciplines, proliferated over the past fifteen years or so. Whilst, using the term ethos, this paper sets out my own view of morality as a field level phenomena readers may find it informative to think of Abend’s notion of the moral background [31]. This comment is particularly aimed at readers of a philosophical disposition who may be reassured by the clear link between Abend’s ideas and Searle’s work on the making of social worlds [32, 33] and, for that matter, with Charles Taylor’s views, particular those presented in Sources of the Self [34].

2 In this paper I speak of both palliative medicine and palliative care. The latter is, of course, a broad field, one that encompasses the former. Arguable, my comments are primarily applicable to palliative medicine, not least due to the cultural exigencies of medicine and its relationship to healthcare more broadly. For example, it is often the case that medical professionals are placed in a de facto position of leadership. Nevertheless, one would hope that my comments can be considered informative for those working within palliative care more generally. Therefore, whilst not considering them precisely synonymous, I make use of both ‘palliative medicine’ and ‘palliative care’ as terms that are intertwined, often switching between them as a matter of inclination and style. One might also note that, whilst palliative care is primarily associated with end of life care, this does not constitute the whole of the field. Certainly palliative medicine and palliative care contribute to improving the quality of life in non-terminal patients. Nevertheless, field’s origins lie within end of life care and this continues to be its mainstay. Thus, it forms the focus of this article.

3 The term ethos is, of course, closely related to that of character. However, as should be clear, my use of the term is as a field level concept. As such it refers to the moral character - order or normative social structure - of a field, cultural domain or institution. As such whilst I occasionally speak of the moral ethos this is, technically speaking, a tautology; it should be understood as having the same meaning as ethos. I have made significant use of the term elsewhere in my work [12].

4 In fairness to Randall and Downie at various points in their various texts they do represent the Hippocratic and Asklepian as compatible with one another. For example, they acknowledge that Cicely Saunders implies as much and proclaim they hold that Hippocratic - or scientific - observation can be combined with Asklepian attention – the hypnotic gaze of the healer or, roughly, empathy and the way in which it can provide comfort to those we empathise with [4]. However, at other points their comments can seem divisive. However, consider their assertion that it is impossible to simultaneously provide both curative and palliative care [4]. This is clearly not the case in the UK and whilst there may be very little overlap in the US, this is due to the way reimbursements provided by insurance companies structure clinical practice, something that is increasingly seen as highly problematic (cf. [35]).

5 Reviewing this article, Dr. Michael Connoly points out that whilst Cicely Saunders founded the modern hospice movement, this was foreshadowed by the work of the Irish Religious Sisters of Charity in the late 1800s and, in the early 1900s, by St Joseph’s Hospice in London. Furthermore, Saunders spent sometime working in the latter institution. My thanks to Dr. Connoly for drawing my attention to these facts.

6 It is, of course, not only the diversity of palliative and curative medicine that the notion of ethos allows us to accommodate. We can also accommodate difference in ethos between medicine and surgery, where no easy appeal to differing millennia old medico-cultural traditions can be made.

7 This may not be quite right. Bureaucratic propriety seems an important part of clinical practice in general. Similarly, if one suspects wrong doing, but has insufficient reason for making any formal report, one might undertake some form of inquisitorial propriety whilst the aftermath of wrongdoing might result in a need for restorative propriety. However, the practices do not seem to take on any distinctive or novel features as a result of being placed in the context of palliative care, as opposed to any other form of clinical endeavor. This is, arguable, not the case in regards fiduciary and collegial propriety.

8 Whilst I do not intend to make use of the term in this essay I have, elsewhere, discussed these issues in terms of ethos and eidos [12].

9 A meticulous approach to grammatical correctness would demand that, rather than speaking of ethical leadership in palliative medicine, we ought to discuss ethics leadership. Strictly speaking, the former refers to leadership that is done in accordance with the relevant ethics, whilst the latter refers to leadership in matters of ethics. However, not only do I find constructions such as ‘ethics leadership’ aesthetically displeasing, I also think the notion that one risks conflating two different things and confusing the reader to be somewhat ungenerous. Although the following discussion of professionalism might be considered as representing an ethics of leadership, I do not explicitly comment on such ethics. Thus I prefer to continue to use the term ethical leadership when speaking of leadership in ethical matters.

10 At the time of writing the UK medical profession is engaged in an informal discussion regarding the use of CPR on dying or terminally ill patients. Given the overall success rates for the procedure it would seem that it is relatively futile to attempt resuscitation on someone who is frail from old age, or dying due to the advanced stage of their terminal illness. Part of the problem here is that healthcare professionals are not allowed to place DNAR orders on patients without discussing it with them or their families, who often do not understand the intense physical nature of the procedure or the possibilities for success. Understandably, discussion of these matters is not easy, and sometimes the conversation is either avoided or truncated. As a result, despite UK doctors having wide latitude in regards refusing to providing treatments that they consider to be unwarranted or of little to no benefit to the patient, CPR is often provided in instances where the possibility of success is entirely minimal.

11 And, one might add, the nature of objectivity. On this point see Daston and Galison’s history of objectivity, [36] whose analysis of the scientific point of view has informed my approach to the issue of ethical expertise and the ‘medical ethical’ point of view [37].
